# Bacteriologic profile and antimicrobial resistance in infants aged 1 year or younger with congenital nasolacrimal duct obstruction

**DOI:** 10.1007/s10384-025-01197-0

**Published:** 2025-04-26

**Authors:** Zhansaya Sultanbayeva, Botagoz Issergepova, Aida Kapanova, Kairat Ruslanuly

**Affiliations:** 1 First Ophthalmology Department, Kazakh Eye Research Institute, Almaty, Kazakhstan; 2 Science Management Department, Kazakh Eye Research Institute, Tole Bi Street 95a, Almaty, 050012 Kazakhstan; 3 Kazakhstan Medical University, Almaty, Kazakhstan

**Keywords:** Antimicrobial drug resistance, Infant, Lacrimal duct obstruction, Microbial sensitivity tests

## Abstract

**Purpose:**

The aim of the study was to evaluate conjunctival flora and antibiotic susceptibility in infants aged 1 year or younger with congenital nasolacrimal duct obstruction (CNLDO), creating an overall profile of antimicrobial susceptibility.

**Study design:**

Retrospective.

**Methods:**

The analysis was conducted at the Kazakh Eye Research Institute over a period of 6 years, from January 2017 to December 2022. Cultures were grown on various agars for bacterial and fungal analyses, with sensitivity testing via Vitek 2 Compact.

**Results:**

We examined 1210 conjunctival cultures from infants with CNLDO, yielding 1212 isolates. Most were gram-positive bacteria (77.15%), with fewer gram-negative bacteria (22.28%) and fungi (0.57%). Among the gram-positive bacteria, *Staphylococcus* species (61.06%) were predominant, including *S epidermidis* (17.49%), *S aureus* (10.73%), and *S saprophyticus* (9.32%). *Enterococcus* species (6.52%) and *Streptococcus* species (6.02%) followed. Among the gram-negative bacteria, *Escherichia coli* (5.78%) was most prevalent, followed by *Pseudomonas* species. (4.54%), *Enterobacter cloacae* (3.71%), and *Klebsiella* species (3.63%). The majority of the fungi were *Candida albicans*, accounting for 4 isolates (0.33%). Most of the bacteria showed high sensitivity to moxifloxacin (92.52%), levofloxacin (88.99%), gentamicin (86.74%), vancomycin (86.52%), cefotaxime (85.27%), and ofloxacin (85.62%). High resistance was noted for erythromycin (32.84%), clindamycin (28.13%), and tetracycline (21.65%).

**Conclusion:**

In this study, we identified *Staphylococcus*, *Enterococcus*, and *Streptococcus* species and *E coli* as key CNLDO bacteria and highly responsive to antibiotics like levofloxacin and moxifloxacin. These findings guide effective antibiotic choices for CNLDO treatment, aiding in the prevention of antibiotic resistance.

**Supplementary Information:**

The online version contains supplementary material available at 10.1007/s10384-025-01197-0.

## Introduction

Congenital nasolacrimal duct obstruction (CNLDO) is a prevalent cause of tearing in newborns and infants aged up to 1 year, with an estimated prevalence of 11–20% among this age group [1–3]. In most cases, the treatment for CNLDO is conservative, including methods like lacrimal sac massage, eyelid care, and use of antibacterial drops [4–6]. However, a debate about the role of antibiotic therapy in treating this condition is ongoing [7]. Notably, guidelines from various countries present differing recommendations on the use of antibiotics in CNLDO. For instance, the Polish and Japanese CNLDO guidelines recommend that antimicrobial eye drops should primarily be used when evidence of infection spreading exists or when significant eye discharge is present, emphasizing a cautious approach to avoid unnecessary antibiotic use [8, 9]. This need for caution contrasts with practices in other regions where antibiotics may be more liberally applied, even without clear signs of infection. Such variations highlight the importance of considering local bacterial resistance patterns and health care practices when prescribing treatment [10].

The primary concern with untreated CNLDO is the progression to a chronic stage, potentially leading to recurrent bacterial infections and inflammation in the eye's accessory structures [3, 11]. Moreover, in infants, whose immune systems are still developing, improper use of antibiotics can accelerate the development of bacterial resistance [12–14]. Consequently, selecting the appropriate antibacterial drugs sometimes requires antibiotic sensitivity testing [8, 15, 16]. Understanding the conjunctival flora in infants with CNLDO is therefore crucial when administering empiric antibiotic therapy during their first year of life [12, 17].

This study focused on analysis of the distribution of conjunctival flora in infants aged 1 year or younger with CNLDO, thereby creating an overall profile of antimicrobial susceptibility.

## Patients and methods

The study was approved by the local ethics committee of Kazakh Eye Research Institute, Almaty, Kazakhstan and conducted in accordance with the Declaration of Helsinki (№1-2024). A retrospective analysis of data from the bacteriologic studies of the conjunctival sacs of children aged up to 1 year, inclusively, and diagnosed with CNLDO was conducted at Kazakh Eye Research Institute from January 2017 to December 2022. Patients with previous lacrimal duct interventions and use of topical antibiotics were excluded.

### Microbiologic analysis

The diagnosis of CNLDO was based on the reflux of mucous or mucopurulent material from the lacrimal point after pressing on the lacrimal sac and was confirmed by subsequent probing.

Conjunctival culture sampling was performed according to the standard procedure for culturing samples from the conjunctival sac. The collected samples were cultured on plates containing 5% blood agar, Endo agar, and salt agar (CHROMagar). For fungal cultures, Sabouraud agar (Microgen) was used. Antimicrobial sensitivity testing was performed by use of an automated antimicrobial susceptibility testing system (Vitek 2 Compact; bioMérieux). All the isolates were categorized into gram-positive and gram-negative bacteria (further subcategorized according to species) and into fungi.

### Statistical analysis

Statistical analysis was performed using the GraphPad Prism 10 software program (GraphPad Software). As all the data were categorical variables, they were expressed as numbers, rates, and percentages.

For analytic purposes, the study participants were divided into 2 groups: those aged 1 year and those aged younger than 1 year. This division was made based on the assumption that the bacterial flora might differ in the 2 age groups and might affect the choice of antibiotic. The Fisher exact test was used to look for statistical associations between the age groups and the type of microorganisms isolated.

## Results

Over a 6-year period, conjunctival culture sampling of 1210 eyes from infants aged 1 year or younger with CNLDO was examined: 2017, 15 eyes; 2018, 553 eyes; 2019, 376 eyes; 2020, 25 eyes; 2021,112 eyes; 2022, 129 eyes. At the end of 2017, we acquired equipment for bacterial culturing, which enabled us to begin this study. The decrease in the number of patients in 2020 was due to the COVID-19 pandemic.

The study’s findings showed that gram-positive bacteria were detected in 77.15% of isolates (935 isolates), gram-negative bacteria in 22.28% of isolates (270 isolates), and fungi in 0.57% of isolates (7 isolates). The distribution of isolates is presented in Supplemental Table 1.

Among the gram-positive bacteria (77.15%, 935 isolates), *Staphylococcus* species (61.06%, 740 isolates) were commonly encountered, with *S epidermidis* (17.49%, 212 isolates), *S aureus* (10.73%, 130 isolates), and *S saprophyticus* (9.32%, 113 isolates) being the most prevalent. This was followed by *Enterococcus* species (6.52%, 79 isolates), including *E faecalis* (2.89%, 35 isolates) and *E faecium* (0.83%, 10 isolates). Subsequently, *Streptococcus* species (6.02%, 73 isolates) were observed less frequently, specifically *S agalactiae* (2.56%, 31 isolates), *S mitis* (0.99%, 12 isolates), and *S oralis* (0.66%, 8 isolates).

Among the gram-negative bacteria (22.28%, 270 isolates), *E coli* (5.78%, 70 isolates) was the most prevalent, followed by *Pseudomonas* species (4.54%, 55 isolates), particularly *P aeruginosa* (3.96%, 48 isolates). *Klebsiella* species (3.63%, 44 isolates), with *K oxytoca* (1.90%, 23 isolates), and *Enterobacter cloacae* (3.71%, 45 isolates) were also notable. *Neisseria* species (0.99%, 12 isolates) were less frequently observed.

Among the 7 fungal isolates identified (0.57%), the majority were *Candida albicans*, accounting for 4 isolates (0.33%).

The prevalence of gram-positive bacteria differed significantly between patients aged 1 year and those aged younger than 1 year (*P* = 0.0261; Table 1). For the fungal isolates, the number was very small (0.57%, only 7 in total), so no statistical conclusion can be drawn from these data alone.

**Table 1:** Comparison of bacteria profile of 2 subgroups demarcated by 1 year of age

Age, y	No. of isolates	Gram staining		Fungus
		Positive	Negative	
< 1	671	502	166	3
> 1	541	433	104	4
Overall	1212	935	270	7
*P* value	NA	0.0261*		NA

*NA* not applicable

*P* value for Fisher exact test

^*^Significance

**Fig. 1:**
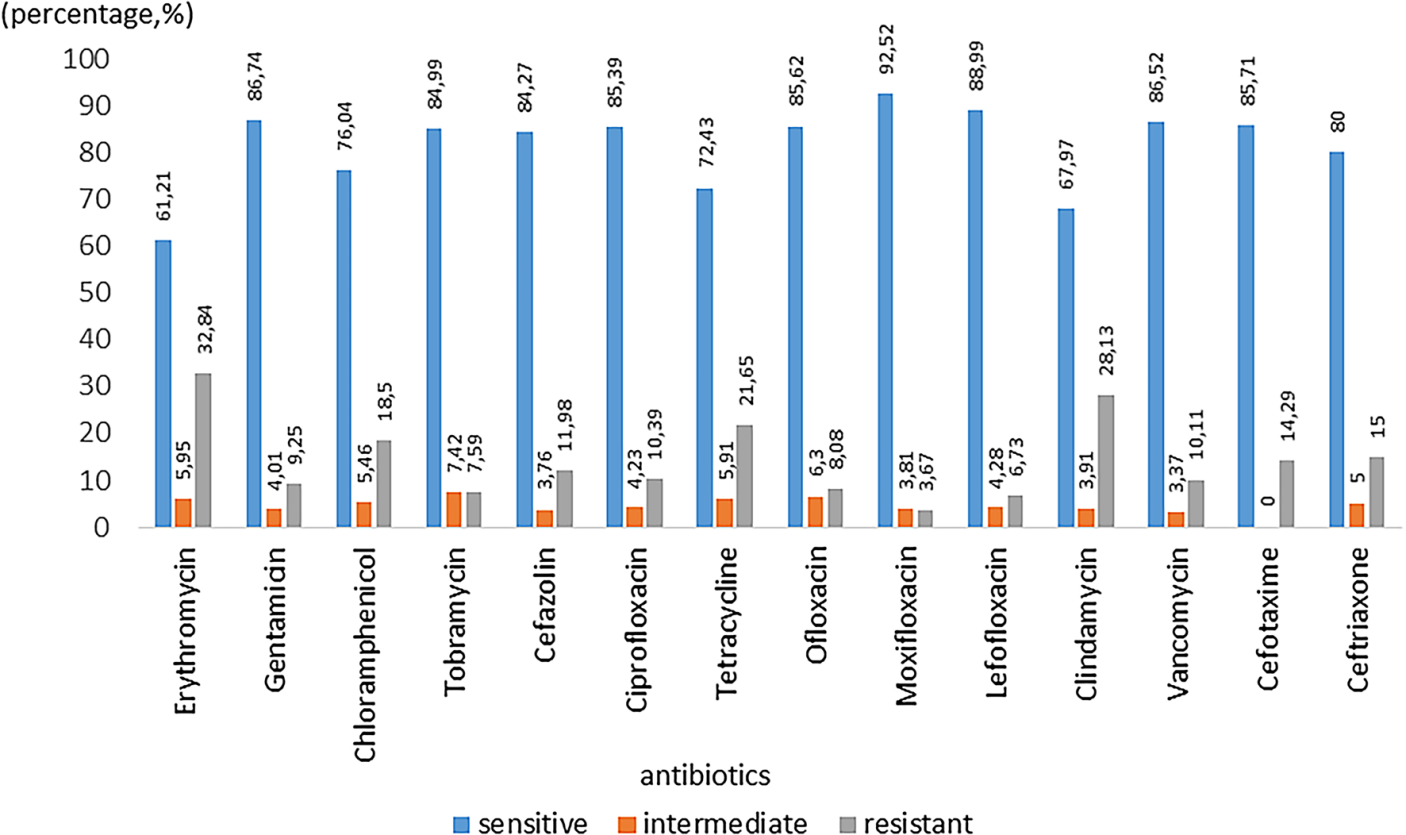
Overall antibiotic sensitivity and resistance pattern chart of all isolated bacterial strains (N = 1212) against different antibiotics

Most of the bacteria were highly sensitive to moxifloxacin (92.52%), levofloxacin (88.99%), gentamicin (86.74%), vancomycin (86.52%), cefotaxime (85.27%) and ofloxacin (85.62%). A high degree of resistance to erythromycin (32.84%), clindamycin (28.13%), and tetracycline (21.65%) was observed (Fig. 1).

We also individually studied the antibiotic sensitivity of each bacterium and provide it in Supplemental Material 2.

## Discussion

In our center, we used conjunctival sampling to minimize discomfort and potential risks for the infant population involved in this study. This noninvasive approach is both ethically and clinically appropriate for young children. In the present study, cultures were obtained from conjunctival samples rather than from mucopurulent material from the lacrimal point. Whilst mucopurulent material may provide direct insights, we relied on conjunctival samples owing to their established reliability in previous studies [8, 9]. Specifically, the literature has shown that the microbial flora obtained via conjunctival swabs correlates strongly with that of lacrimal sac secretions, supporting the validity of this approach.

The literature review showed only 2 studies specifically addressing the microflora in children aged 1 year or younger with CNLDO, which we will reference in our discussion [12, 17].

Our study found a higher prevalence of gram-positive bacteria (77.15%) when compared with other studies, which reported ranges from 60.5 to 72%. Conversely, the prevalence of gram-negative bacteria in our study (22.28%) was slightly lower than the ranges reported in other studies, which varied from 33 to 39.5%. Additionally, we detected fungi in 0.57% of the isolates, whereas other studies did not report any fungi isolates.

Among the gram-positive bacteria in our study, *Staphylococcus* species were the most common, accounting for 61.06% of the isolates. This prevalence is significantly higher than those of other studies, which reported a range of 5.3–26%. However, *Streptococcus* species were less frequently observed in our study (6.02%) than in other studies, which reported ranges from 31 to 50%. *Enterococcus* species were not reported in the other studies we reviewed.

Among the gram-negative bacteria, the prevalence of *Pseudomonas* species (4.54%) in our study is consistent with those of other studies, which reported a range of 0–4%. *Neisseria* species appeared in 0.99% of our isolates, comparable to the ranges of 0–13.2% found in other studies. However, *Enterobacter* species, *Klebsiella* species, and *E coli* were not reported in other studies. This discrepancy may be explained by differences in the cultivation methods used in each report, as well as by demographic factors such as ethnicity and geographic location, which can influence the microbiologic profile of congenital nasolacrimal duct obstruction in young children.

Considering that these were infants who had not previously received any antibiotic therapy for their eyes, we decided to compare our results with those of the most recent available study on antibiotic sensitivity in children aged 1 year or younger, which was conducted 4 years ago by Zheng and colleagues [17]. Both studies reported high sensitivity to gentamicin and vancomycin (86.74% [n = 973] and 86.52% [n = 89] in our study compared to 100% (n = 19, in both cases). Regarding fluoroquinolone antibiotics, our study showed high sensitivity to moxifloxacin (92.52%, n = 682), levofloxacin (88.99%, n = 327), ofloxacin (85.62%, n = 953), and ciprofloxacin (85.39%, n = 1136), as compared with 100% (n = 10), 84% (n = 25), 87.5% (n = 8), and 100% (n = 2), respectively, in their study. They also reported notable resistance levels for erythromycin (42.1%, n = 19), clindamycin (15.38%, n = 13), and tetracycline (45.45%, n = 11), as compared with our findings of 32.84% (n = 1142), 28.13% (n = 128), and 21.65% (n = 1150), respectively. These comparisons indicate that the antibiotic sensitivity and resistance patterns observed in our study are largely consistent with those reported by our colleagues. Unfortunately, whether systemic antibiotics were previously prescribed is unknown, which could have affected the sensitivity results of the ocular flora.

Our study demonstrated a high level of positive culture results in children with CNLDO. Several studies have been conducted on the microbiology of CNLDO, and the bacterial spectrum varies depending on the individual’s age and changes over time [11–18]. Several issues might have affected the antimicrobial resistance in our study: the lack of legislative regulation regarding the sale of antibacterial drops allows anyone to purchase them without restrictions. This can lead to misuse and overuse, contributing to the development of resistance. The clinical protocol in Kazakhstan includes the prophylactic application of 1% tetracycline ointment in the eyes of newborns, performed at the end of the first hour after birth. Previous studies on bacterial strains associated with CNLDO had broader age ranges and limited sample sizes, and some have become outdated. Regional variations must be considered to optimize antibiotic use and reduce resistance.

Our findings support the recommendations of Vagge and colleagues, who emphasized the importance of tailoring antibiotic therapy on the basis of microbial sensitivity profiles and clinical indications rather than on the basis of routine prophylaxis [3]. By identifying the prevalent bacterial strains and their antibiotic susceptibility, our study provides valuable data that can inform more targeted and effective treatment strategies, thereby aligning with global best practices for antimicrobial use. In contrast, some experts advocate for the use of topical antibacterial therapy in cases of CNLDO even in the absence of mucopurulent discharge. However, both the Polish and the Japanese guidelines for CNLDO recommend a more conservative approach, advising the use of antimicrobial eye drops only when a significant risk exists of the infection spreading or when discharge is present.

Our study has some limitations. Its retrospective nature and the fact that it is a single-center study mean our results might not be representative of other regions. In this study, we excluded patients with a history of antibiotic use, which limited our ability to directly compare antibiotic resistance-susceptibility patterns between those who had never used antibiotics and those who had. However, a strong point of our study is that we examined a large number of infants aged 1 year or younger using a standardized method of sample collection from the conjunctival sac. Future studies from different regions of the world are needed to understand the variability of ocular surface microflora in children from different countries.

In conclusion, this study provides significant insights into the distribution of conjunctival flora and antimicrobial susceptibility in infants aged 1 year or younger with CNLDO. Our findings highlight a higher prevalence of gram-positive bacteria, particularly *Staphylococcus* species *(S epidermidis, S aureus, and S saprophyticus)*. Both gram-positive and gram-negative bacteria demonstrated high sensitivity to moxifloxacin, levofloxacin, gentamicin, vancomycin, cefotaxime, and ofloxacin, while showing resistance to erythromycin, clindamycin, and tetracycline, thereby highlighting the necessity for careful selection of empiric antibiotic therapy to prevent the progression of bacterial resistance.

## Supplementary Information

Below is the link to the electronic supplementary material.

Supplementary file1 (XLSX 17 KB)

Supplementary file2 (XLSX 15 KB)
